# Ethylene is involved in strawberry fruit ripening in an organ-specific manner

**DOI:** 10.1093/jxb/ert257

**Published:** 2013-10-05

**Authors:** Catharina Merchante, José G. Vallarino, Sonia Osorio, Irene Aragüez, Natalia Villarreal, María T. Ariza, Gustavo A. Martínez, Nieves Medina-Escobar, Marcos P. Civello, Alisdair R. Fernie, Miguel A. Botella, Victoriano Valpuesta

**Affiliations:** ^1^Instituto de Hortofruticultura Subtropical y Mediterránea (IHSM-UMA-CSIC), Departamento de Biología Molecular y Bioquímica, Facultad de Ciencias, Universidad de Málaga, Campus de Teatinos s/n, E-29071 Málaga, Spain; ^2^IIB-INTECH (CONICET-UNSAM), Instituto de Investigaciones Biotecnológicas-Instituto Tecnológico de Chascomús, Camino de Circunvalación Laguna, Km 6, (B7130IWA) Chascomús, Pcia, Buenos Aires, Argentina; ^3^Facultad de Ciencias Exactas, Universidad Nacional de La Plata (UNLP), 47 y 115, (1900) La Plata, Argentina; ^4^INFIVE (CONICET-UNLP), Instituto de Fisiología Vegetal, Diag. 113 y Calle 61 no. 495 – C.c 327, (1900) La Plata, Argentina; ^5^Max-Planck-Institut für Molekulare Pflanzenphysiologie, Am Mühlenberg 1, D-14476 Potsdam-Golm, Germany

**Keywords:** Ethylene, fruit, metabolic profiling, non-climacteric, ripening, strawberry.

## Abstract

The fruit of the strawberry *Fragaria×ananassa* has traditionally been classified as non-climacteric because its ripening process is not governed by ethylene. However, previous studies have reported the timely endogenous production of minor amounts of ethylene by the fruit as well as the differential expression of genes of the ethylene synthesis, reception, and signalling pathways during fruit development. Mining of the *Fragaria vesca* genome allowed for the identification of the two main ethylene biosynthetic genes, 1-aminocyclopropane-1-carboxylic acid (ACC) synthase and ACC oxidase. Their expression pattern during fruit ripening was found to be stage and organ (achene or receptacle) specific. Strawberry plants with altered sensitivity to ethylene could be employed to unravel the role of ethylene in the ripening process of the strawberry fruit. To this end, independent lines of transgenic strawberry plants were generated that overexpress the *Arabidopsis etr1-1* mutant ethylene receptor, which is a dominant negative allele, causing diminished sensitivity to ethylene. Genes involved in ethylene perception as well as in its related downstream processes, such as flavonoid biosynthesis, pectin metabolism, and volatile biosynthesis, were differently expressed in two transgenic tissues, the achene and the receptacle. The different transcriptional responsiveness of the achene and the receptacle to ethylene was also revealed by the metabolic profiling of the primary metabolites in these two organs. The free amino acid content was higher in the transgenic lines compared with the control in the mature achene, while glucose and fructose, and citric and malic acids were at lower levels. In the receptacle, the most conspicuous change in the transgenic lines was the depletion of the tricarboxylic acid cycle intermediates at the white stage of development, most probably as a consequence of diminished respiration. The results are discussed in the context of the importance of ethylene during strawberry fruit ripening.

## Introduction

The strawberry (*Fragaria×ananassa*, Duch.) is an important crop worldwide, and the potential benefits of its consumption in the prevention of cardiovascular and neurodegenerative diseases, obesity, cancer, and ageing are currently under investigation (Seeram, 2008; Giampieri *et al.*, 2012). Despite the increasing amount of information regarding the processes involved in strawberry fruit ripening (Medina-Escobar *et al.*, 1997; Aharoni and O’Connell, 2002; Bombarely *et al.*, 2010; Csukasi *et al.*, 2011, 2012), little is known about the regulation of these processes, including the role played by the various phytohormones. The main reason for this relatively poor understanding in comparison with other fruits is that what is commonly referred to as the strawberry fruit is in fact a false fruit that is composed of achenes (true fruits that evolve from ovaries), and the engrossed flower receptacle (fleshy part), and both organs are connected through vascular bundles (Perkins-Veazie, 1995). These two organs, the achene and the receptacle, although highly interconnected throughout their developmental programmes, particularly at early developmental stages, are very different in terms of cell ontogeny and function, as has been revealed by both gene expression studies (Aharoni and O’Connell, 2002; Csukasi *et al.*, 2012) and metabolic profiling (Fait *et al.*, 2008). Therefore, the underlying regulatory processes that occur during development and ripening are expected to differ for each organ. Importantly, studies on the whole fruit, without dissection into its two composite parts, mask tissue-specific differences, the knowledge of which is required to draw reliable conclusions.

Ethylene, the simplest olefin, plays a key role in many aspects of the plant life cycle, regulating various biological processes, including seed germination, cell elongation, root initiation, flower development, sex determination, fruit ripening, senescence, and responses to biotic and abiotic stresses (Bleecker and Kende, 2000). Depending on the role of ethylene in ripening, fruits are classified as either climacteric, such as the tomato, apple, banana, and avocado, all of which present increased respiration and a burst of ethylene biosynthesis at the onset of ripening, or non-climacteric, such as the grape and pepper, which show no relevant increase in respiration and ethylene production during ripening (Giovannoni, 2004). In climacteric fruits, ethylene is the key hormone that controls fruit ripening (Giovannoni, 2007; Klee and Giovannoni, 2011; Osorio *et al.*, 2011). In plants, ethylene is synthesized from methionine, and the last two steps in ethylene biosynthesis are the main regulatory control points. These steps consist of the conversion of *S*-adenosyl-l-methionine into 1-aminocyclopropane-1-carboxylic acid (ACC) by ACC synthase (ACS) and the oxidation of ACC to ethylene by ACC oxidase (ACO) (Yang and Hoffman, 1984). Both enzymes are encoded by multigene families in many plant species, with differences in the expression and regulation of the various members (Lin *et al.*, 2009). This pathway has been studied in detail in climacteric tomato fruits, and different family members have been associated with specific developmental stages (Cara and Giovannoni, 2008; Itkin *et al.*, 2009; Yokotani *et al.*, 2009; Kamiyoshihara *et al.*, 2010; Klee and Giovannoni, 2011).

The strawberry fruit has traditionally been classified as non-climacteric, based on its low endogenous production of ethylene compared with standard climacteric fruits and because of the inability to accelerate strawberry fruit ripening by the external application of ethylene or ethylene-releasing compounds (Perkins-Veazie, 1995). However, despite the low levels of this hormone in the strawberry fruit (Leshem and Pinchasov, 2000), recent studies have shown that the situation is not that simple and have generated some controversy in the field (Trainotti *et al.*, 2005; Iannetta *et al.*, 2006). It has been reported that, despite its low concentration, ethylene presents a characteristic pattern of production during different developmental stages; it is relatively high in green fruits, decreases in white fruits, and finally increases at the red stage of ripening (Perkins-Veazie *et al.*, 1996; Iannetta *et al.*, 2006). Interestingly, this last increase is accompanied by an enhanced respiration rate that resembles that which occurs in climacteric fruits at the onset of ripening (Iannetta *et al.*, 2006). In the strawberry, two *ACO* genes (*FaACO1* and *FaACO2*) and three ethylene receptor genes, two type I (*FaEtr1* and *FaErs1*) and one type II (*FaEtr2*), have been identified. Moreover, their expression during ripening as well as their response to different hormone treatments have been studied (Trainotti *et al.*, 2005). In general, a good correlation exists between the expression of all of these genes and ethylene production (Iannetta *et al.*, 2006). However, the expression and the ethylene production data corresponded to the whole berry; that is, the achenes and receptacle (Trainotti *et al.*, 2005).

The external application of ethylene or its precursors, and the application of inhibitors of ethylene synthesis or perception, have been utilized to discern the function of this hormone in the growth and ripening of the strawberry. Although the application of ethylene to strawberry fruits does not have an obvious effect on ripening, it does have an effect on the expression of a subset of ripening-related genes (Trainotti *et al.*, 2001; Castillejo *et al.*, 2004; Bustamante *et al.*, 2009).

Ethylene causes the down-regulation of several cell wall-related genes that are involved in fruit softening, such as β-galactosidase, pectin methylesterase, and β-xylosidase (Trainotti *et al.*, 2001; Castillejo *et al.*, 2004; Bustamante *et al.*, 2009), while the expression of other cell wall-related genes, such as expansin *FaEXP2* (Civello *et al.*, 1999), is ethylene insensitive. Blocking ethylene action with 1-methylcyclopropene (1-MCP) results in the down-regulation of the polygalacturonase gene (Villarreal *et al.*, 2009). In addition to its effects on gene expression, ethylene treatment also decreases the enzymatic activities of endo-1,4-β-glucanase and β-xylosidase (Villarreal *et al.*, 2010). These results indicate that although it is not as relevant as in climacteric fruits, ethylene may play a role in strawberry fruit ripening.

To gain further insight into ethylene production during strawberry ripening, the expression of the key genes involved in ethylene biosynthesis, *ACS* and *ACO*, was analysed in both the achenes and receptacles at different developmental stages. Next, the role of ethylene during strawberry ripening was investigated by generating transgenic strawberry plants with reduced ethylene sensitivity. These plants were generated through ectopic expression of the dominant negative mutant allele of the *Arabidopsis* ethylene receptor gene *etr1-1*, which has been shown to diminish ethylene insensitivity in different plant species, such as *Arabidopsis*, tomato, petunia, tobacco, and birch (Chang *et al.*, 1993; Wilkinson *et al.*, 1997; Vahala *et al.*, 2003; Knoester *et al.*, 2005). Transcriptional and metabolic analyses of the transgenic fruits with respect to organ and developmental specificity revealed different roles for ethylene in the growth and ripening of the achene and receptacle. The results are discussed in terms of the specific development patterns for these two organs, which together form the edible fruit.

## Materials and methods

### Plant material, transformation, and sampling

Strawberry plants (*Fragaria×ananassa* Duch.) were grown in a greenhouse under natural light conditions in southern Spain (Málaga). Transformation of *F.×ananassa* cv. Chandler plants was performed according to the protocol described by El-Mansouri *et al.* (1996). Strawberry leaf discs were transformed with *Agrobacterium tumefaciens* LBA4404 carrying a pCD-2 plasmid that contained the kanamycin resistance gene *nptII* and the cDNA for the mutant allele of *etr1-1* (At1g66340) in the sense orientation under the control of a single constitutive [*Cauliflower mosiac virus* (CaMV) *35S*] promoter (Wilkinson *et al.*, 1997). All the *F.×ananassa* plants were vegetatively propagated each season using stolons. The plants used in this work corresponded to the first, second, and third vegetative generations, and the transgenic lines were evaluated in consecutive years.

Fruits from wild-type and transgenic plants were harvested at three different developmental stages: green fruit (G; green achenes and receptacle), white fruit (W; green achenes and white receptacle), and red fruit (R; red achenes and receptacle), corresponding to 12, 21, and 35 d post-anthesis, respectively, as previously described by de la Fuente *et al.* (2006). Prior to removing the achenes from the receptacle, whole fruits were collected and immediately frozen in liquid nitrogen to avoid changes in gene transcription due to wounding. Then, for the achene and receptacle analysis, the achenes of G, W, and R fruits were carefully removed from the corresponding receptacles with a scalpel tip. Analyses of the fruits were performed on a minimum of four or five separate pools of 30 fruits each for each ripening stage. Each pool was from one individual plant.

Fruit firmness was measured using a penetrometer with 3mm^2^ surface needles. Three punctures were made in opposite sites per fruit. Between 20 and 25 fruits per line were used.

### Metabolome analysis

Metabolite extraction, derivatization, standard addition, and sample injection for gas chromatography–mass spectrometry (GC-MS) were performed according to Osorio *et al.* (2012). The mass spectra were cross-referenced with those in the Golm Metabolome database (Kopka *et al.*, 2005). The content of each metabolite was determined by normalizing the integration area of a characteristic fragment ion trace to the integration area of the internal standard, ribitol (*m/z* 319), and the fresh weight of the plant material was extracted.

### Total phenolics

Total phenolics were determined according to the Folin–Ciocalteu procedure (Waterhouse, 2001). Total phenolic content was expressed as gallic acid equivalents (GAE) in milligrams per 100g of fresh weight of achenes.

### RNA extraction and quantification for qRT–PCR

The RNA extraction and quantitative reverse transcription–PCR (qRT–PCR) were performed as described by Osorio *et al.* (2008). The expression of the genes encoding various ethylene receptors (*etr1-1*, *FaETR1*, *FaETR2*, and *FaERS1*), phenylalanine ammonia lyase (*FaPAL*), chalcone synthase (*FaCHS*), 1-aminocyclopropane-1-carboxylate oxidase 1 (*FaACO1*), 1-aminocyclopropane-1-carboxylate oxidase 2 (*FaACO2*), 1-aminocyclopropane-1-carboxylate oxidase 3(*FaACO3*), 1-aminocyclopropane-1-carboxylate synthase 1 (*FaACS1*), 1-aminocyclopropane-1-carboxylate synthase 2 (*FaACS2*), 1-aminocyclopropane-1-carboxylate synthase 3 (*FaACS3*), 1-aminocyclopropane-1-carboxylate synthase 4 (*FaACS4*), pectin methyl esterase (*FaPE1*), pectate lyase A (*FaPLA*), polygalacturonase 1 and 2 (*FaPG1* and *FaPG2*), *O*-methyltransferase (*FaOMT*), quinone reductase (*FaQR*), MYB transcription factors (*FaMYB1* and *FaMYB10*), and d-galacturnonate reductase (*FaGalUR*), was analysed by real-time qRT–PCR using the fluorescent intercalating dye EvaGreen in an iCycler detection system (Bio-Rad). Relative quantification of the target expression level was performed using the comparative Ct method (Pfaffl, 2001). To normalize gene expression for differences in the efficiency of cDNA synthesis, the transcript levels of the constitutively expressed glyceraldehyde-3-phosphate dehydrogenase gene (*GAPDH*) were measured (Opazo *et al.*, 2010; Salvatierra *et al.*, 2010). The primers used are shown in Supplementary Table S1 available at *JXB* online.

### Phylogenetic analysis

Sequence analysis was performed with ClustalW, and the Neighbor–Joining method was used to generate the tree. The percentages of replicate trees in which the associated proteins clustered together in the bootstrap test (1000 trials) are shown next to the branches. The trees are drawn to scale, with branch lengths in the same units as those of the evolutionary distances used to infer the phylogenetic tree. The evolutionary distances were computed using the *p*-distance method, and the scale is the number of amino acid differences per site. The evolutionary analyses were conducted in MEGA5 (Tamura *et al.*, 2011).

### Accession numbers

Sequence data from this article can be found at http://www.strawberrygenome.org under the following accession numbers: gene 31839 (*FvACS1*), gene 19023 (*FvACS2*), gene 30682 (*FvACS3*), gene 11392 (*FvACS4*), gene 01202 (*FvACO1*), gene 19733 (*FvACO2*), and gene 11261 (*FvACO3*). Other sequence data can be found in GenBank under the following accession numbers: AAT77723 (*FaACS1*), AAF97614 (*SlACS1A*), AAF97615 (*SlACS1B*), AAP96918 (*SlACS2*), AAA78789 (*SlACS3*), P29535 (*SlACS4*), AAK72430 (*SlACS5*), AAK72433 (*SlACS6*), AAC32317 (*SlACS7*), AAK72431 (*SlACS8*), CAH65482 (*FaACO1*), CAH65483 (*FaACO2*), ADO32577 (*SlACO1*), Y00478 (*SlACO2*), Z54199 (*SlACO3*), BAA34924 (*SlACO4*), AJ715790 (*SlACO5*), AB201756 (*FaPAL*), AB360390 (*FaCHS*), AY324809 (*FaPE1*), AF339025 (*FaPLA*), AF280662 (*FaPG1*) and AF380299 (*FaPG2*), AJ297511 (*FaETR1*), AJ297513 (*FaETR2*), AJ297512 (*FaERS1*), EU155162 (*FaMYB10*), AF401220 (*FaMYB1*), AF220491 (*FaOMT*), AY158836 (*FaQR*), and AF039182 (*FaGalUR*).

### Statistical analysis

Statistical analysis was performed with SPSS Statistics v20 (IBM Corp.) and the R environment (http://www.r-project.org) using analysis of variance (ANOVA). The means were compared by Student’s *t*-test and Tukey HSD for metabolite and expression analysis, respectively. Correlation analysis based on Pearson correlation was performed using R software (Ihaka and Gentleman, 1996).

## Results

### Expression of genes encoding ethylene biosynthesis enzymes in strawberry fruits display a tissue-/stage-specific pattern

Previous studies of ethylene production in strawberry fruits have been performed in the so-called fruits, which are a combination of both the achenes and the receptacle. However, this type of analysis provides ambiguous information due to the heterogeneity of the sample. To be consistent with the previous literature, the term ‘fruit’ has been used to describe the complete berry, but a distinction is made between the receptacles and achenes when they are studied separately.

As previously mentioned, ethylene production has been measured in strawberry fruits during ripening. However, the focus of the present investigation was the independent ethylene production of the achenes and receptacles. It is important to note that this is technically difficult because ethylene should be produced after the physical damage resulting from the separation of the achenes. A reasonable correlation between the transcription of the genes encoding ACS and ethylene production has, however, been reported (Lin *et al.*, 2009). Therefore, it was decided to analyse the expression of *ACS* and *ACO* in the achenes and receptacles separately. In the strawberry, one *ACS* (*FaACS1*, GenBank accession no. AY661301.1) and two *ACO* genes have been reported (*FaACO1* and *FaACO2*; GenBank accession nos CAH65482 and CAH65483; Trainotti *et al.*, 2005). Previous studies have shown that the diploid *F. vesca* genome contributes to the octoploid genome of the strawberry *F.×ananassa* (Rousseau-Gueutin *et al.*, 2009) with an extremely high sequence identity (Bombarely *et al.*, 2010). Using the available sequence of the wild strawberry *Fragaria vesca* genome (Shulaev *et al.*, 2011), three new *ACS* genes (*FvACS2*, *FvACS3*, *FvACS4*) and one new *ACO* gene (*FvACO3*) were identified, in addition to those that have been previously reported in *F.×ananassa*. Therefore, the *F. vesca* sequences were used to design gene-specific primers for the members of the *FaACS* and *FaACO* gene families that were not previously reported in *F.×ananassa*.

Expression analyses using qRT–PCR were performed separately in the achenes and receptacles at three developmental stages, namely green, white, and red, based on the colour of the receptacle (Supplementary Fig. S1 at *JXB* online). At the green stage, the seed in the achene has reached the cotyledon stage, and the subsequent main events include preparation for the dormancy of the seed and changes associated with the ripening of the dry pericarp (Hollender *et al.*, 2012). In the receptacle, the main change between the green and white stages is cell enlargement, and those between the white and red stages are cell wall disassembly and metabolism associated with ripening (Rose and Bennett, 1999; Brummell, 2006; Fait *et al.*, 2008; Zhang *et al.*, 2011).

**Fig. 1.**
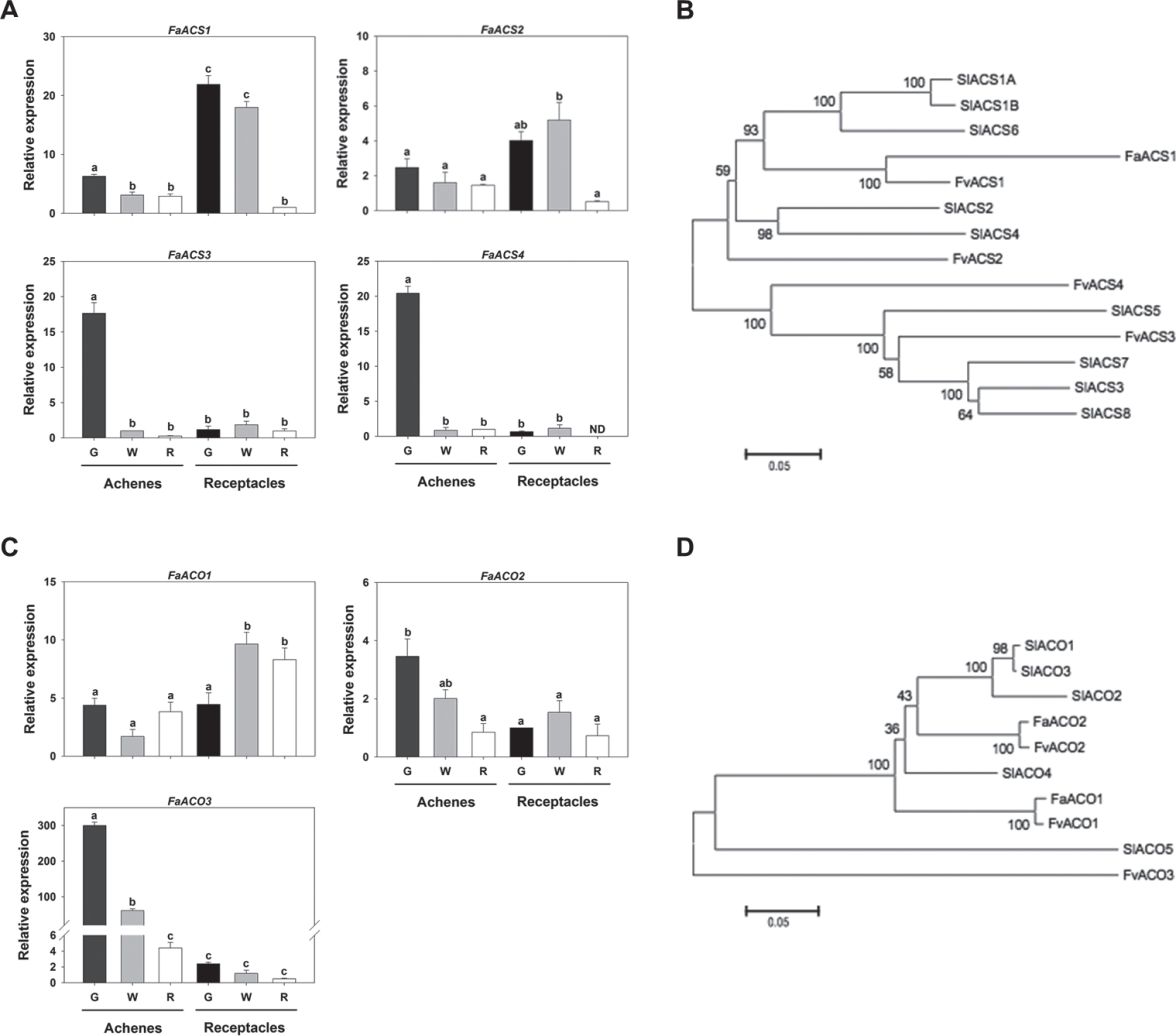
Analysis of expression of the genes encoding ACC synthase (*ACS*) and ACC oxidase (*ACO*) in the strawberry *Fragaria×ananassa*. (A and C) Expression of *ACS* and *ACO*, as determined by qRT–PCR, in the achenes and receptacles of strawberry fruits at three developmental stages (G, green; W, white; R, red). (B and D) Unrooted phylogenetic tree of the ACS and ACO proteins of *F.×ananassa* (Fa), *F. vesca* (Fv), and *Solanum lycopersicum* (Sl). Different letters indicate significant differences, in achenes and receptacles, for each gene using ANOVA and the Tukey HSD test adjusted to a 95% significance level.

The expression of *FaACS1* and *FaACS2* was higher in the green and white receptacles than in any other tissue/stage, whereas the expression of *FaACS3* and *FaACS4* was mostly limited to the green achenes (Fig. 1A). A phylogenetic tree of the four *F. vesca* and *F. ananassa* ACS enzymes with the tomato ACS enzymes revealed that FvACS1 and FaACS1 were grouped with the tomato ACS enzymes involved in fruit ripening (SlACS1, SlACS2, SlACS4, and SlACS6) (Cara and Giovannoni, 2008). FvACS2 was also grouped, at a greater distance, with the same tomato ACS. However, FvACS3 and FvACS4 were grouped apart from the other tomato ACS proteins (Fig. 1B). Regarding the *FaACO* genes, the highest expression of *FaACO1* occurred in the white and red receptacles, while *FaACO2* and *FaACO3* showed the highest expression in the green achenes (Fig. 1C). Their comparison with the corresponding tomato proteins and the generation of a phylogenetic tree showed that FaACO2 and FvACO2 grouped closely with the fruit-specific SlACO1, SlACO3, and SlACO4 (Cara and Giovannoni, 2008), while FaACO1 and FvACO1 grouped with the same tomato enzymes at a greater distance. FvACO3 was found to be less closely related to the tomato ACO proteins (Fig. 1D). Overall, the expression patterns of *FaACS* and *FaACO* are indicative of two organ-specific stages, with the highest expression values in the green achenes (*FaACS3*, *FaACS4*, and *FaACO3*) and the green/white receptacles (*FaACS1*).

### Strawberry plants overexpressing *etr1-1* from *Arabidopsis thaliana* have a diminished sensitivity to ethylene

**Fig. 2.**
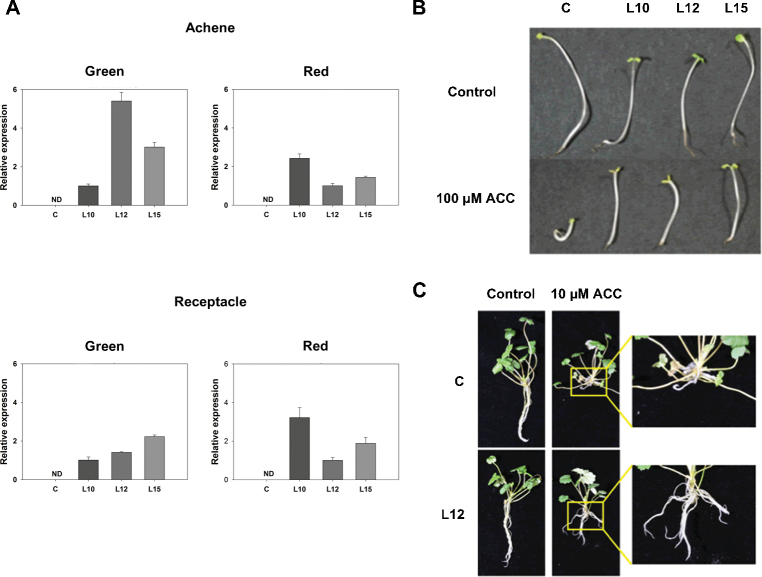
Expression of the *Arabidopsis etr1-1* gene in the fruits of the transgenic lines and analysis of the lower sensitivity of these lines to ethylene. (A) Relative expression of *etr1-1*, as determined by qRT–PCR, in the achenes and receptacles of the green and ripe stages of the transgenic lines (L10, L12, and L15) and control (C). (B) Photographs of control and *etr1-1* plantlets obtained after germination of achenes in normal medium (MS) and medium supplemented with 100 μM ACC. (C) Photographs of 4-week-old *in vitro* control and L12 plants grown in standard (N30K) medium and medium supplemented with 10 μM ACC.

The generation of transgenic strawberry plants displaying reduced ethylene sensitivity was instrumental in this work to investigate the role of ethylene during fruit ripening. Strawberry leaf discs were transformed with a construct containing the *etr1-1* gene under the control of the CaMV *35S* promoter. This construct has been shown to confer ethylene insensitivity in heterologous plant species (Wilkinson *et al.*, 1997). The leaves of 32 independent lines that were resistant to antibiotic selection were analysed for the expression of the transgene. Most of the selected lines expressed the *etr1-1* gene (Supplementary Fig. S2 at *JXB* online). The primers were specific for the transgene. Therefore, no expression was detected in the non-transformed control plants. Three lines that displayed high expression of the transgene in the leaves were selected for further analysis (lines 10, 12, and 15). Next, it was confirmed that the transgene was expressed in the achenes and receptacles of the green and red stages using qRT–PCR. As shown in Fig. 2A, the expression of the transgene was dependent on the tissue and developmental stage, with a relatively good correlation in the expression levels between the achenes and receptacles in the three lines (Fig. 2A). It was previously reported that the *35S* promoter was effective in driving gene expression in strawberry fruits (Agius *et al.*, 2005). Here, it can be added that this promoter is functional in both the achenes and the receptacle.

During the generation of the primary transformants, there was evidence of low sensitivity to ethylene as the transgenic lines showed improved growth *in vitro* compared with the control lines. This is most probably due to ethylene accumulation in the culture flask (data not shown). The effect of increasing the ethylene production in plants grown *in vitro* by the addition of ACC, which is readily converted to ethylene by the action of ACO, was therefore investigated. As shown in Fig. 2C, while 10 μM ACC inhibited the root growth of the *in vitro* control plants, the roots of the transgenic lines exhibited noticeable growth. Next, the decreased sensitivity of the transgenic lines to ethylene was investigated by observing the ethylene-mediated effects on the hypocotyl and root phenotype. As shown in Fig. 2B, the control and transgenic seedlings showed similar growth under the control conditions. However, in the presence of 100 μM ACC, while the control line showed the characteristic triple response [i.e. the inhibition of stem elongation, radial swelling of the stem, and absence of a normal geotropic response (Guzmán and Ecker, 1990)], the transgenic seedlings displayed reduced sensitivity. It is important to note that the concentration required to induce the triple response was unusually high compared with that used in other species (Smalle *et al.*, 1997; Lohar *et al.*, 2009).

### Reduced ethylene sensitivity alters strawberry fruit ripening

**Fig. 3.**
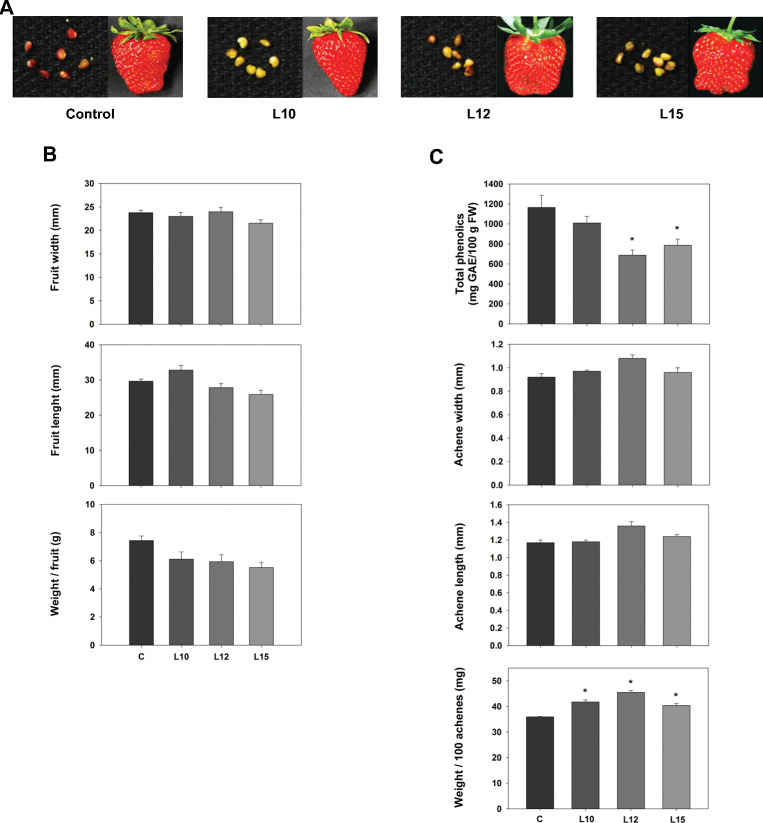
Phenotypic analysis of the ripe fruits of the control and *etr1-1* transgenic plants (L10, L12, and L15). (A) Photographs of the ripe fruits and achenes of the control and *etr1-1* transgenic lines. (B) Width, length, and weight of the ripe fruits. (C) Total phenolics. Width, length, and weight of the ripe achenes. Asterisks indicate differences at a 95% significance level using ANOVA and Tukey HSD as a post-hoc test.

The expression of *etr1-1* in the tomato and petunia causes extreme phenotypes, with tomato fruits remaining unripened for >3 months and petunias showing an extended flower life (Wilkinson *et al.*, 1997). In contrast, the flowers and fruits produced by the three transgenic lines used here did not show any apparent visible differences in colour or size compared with the control at the developmental stages analysed (Fig. 3A). This result was corroborated by statistical analyses of the fruits in terms of width, length, and weight (Fig. 3B). However, differences in the colour of the achenes between the control and transgenic lines were found in ripe fruits. As shown in Fig. 3A, while the achenes showed a dark red colour in the control fruits, they displayed a green or light red colour in the transgenic lines. The content of phenolics was lower in these transgenic lines compared with the control, being significant in two of the lines (Fig. 3C). The achene size and weight were also determined at the fully ripe stage. The *etr1-1* transformants exhibited a significantly higher weight, which was consistent across all three transgenic lines (Fig. 3C). In contrast, the achene size was unaltered (Fig. 3C).

**Fig. 4.**
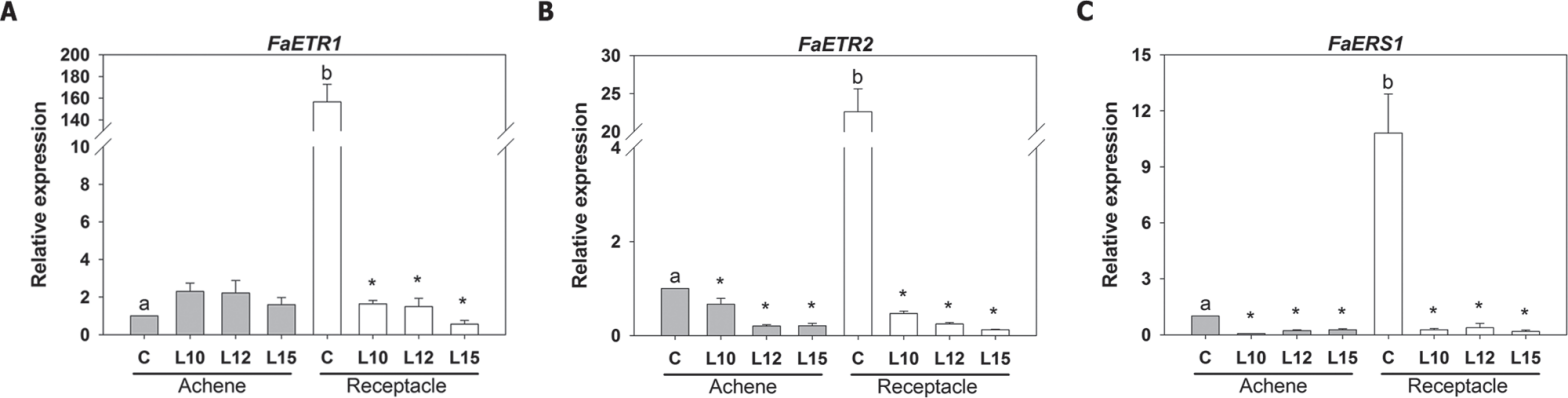
Relative expression of ethylene receptor genes (*FaETR1*, *FaETR2*, and *FaERS1*), as determined by qRT–PCR, in the achenes and receptacles of ripe strawberry fruits. Asterisks indicate significant differences between the transgenic lines and the control for each sample using ANOVA and the Tukey HSD test adjusted to a 95% significance level. Different letters indicate significant differences within the control lines (achene and receptacle) for each gene using ANOVA and the Tukey HSD test adjusted to a 95% significance level.

In strawberry fruits, the expression of the three ethylene receptors as well as their response to external ethylene have been studied during fruit ripening (Trainotti *et al.*, 2005). The expression of these genes during the ripening of the achenes and receptacle was therefore analysed. The results in ripe fruits indicate that the expression of the three genes is much higher in the receptacle than in the achene, and, interestingly, they are down-regulated in the *etr1-1* transgenic lines (Fig. 4A–C). This effect was dramatic in the receptacle.

**Fig. 5.**
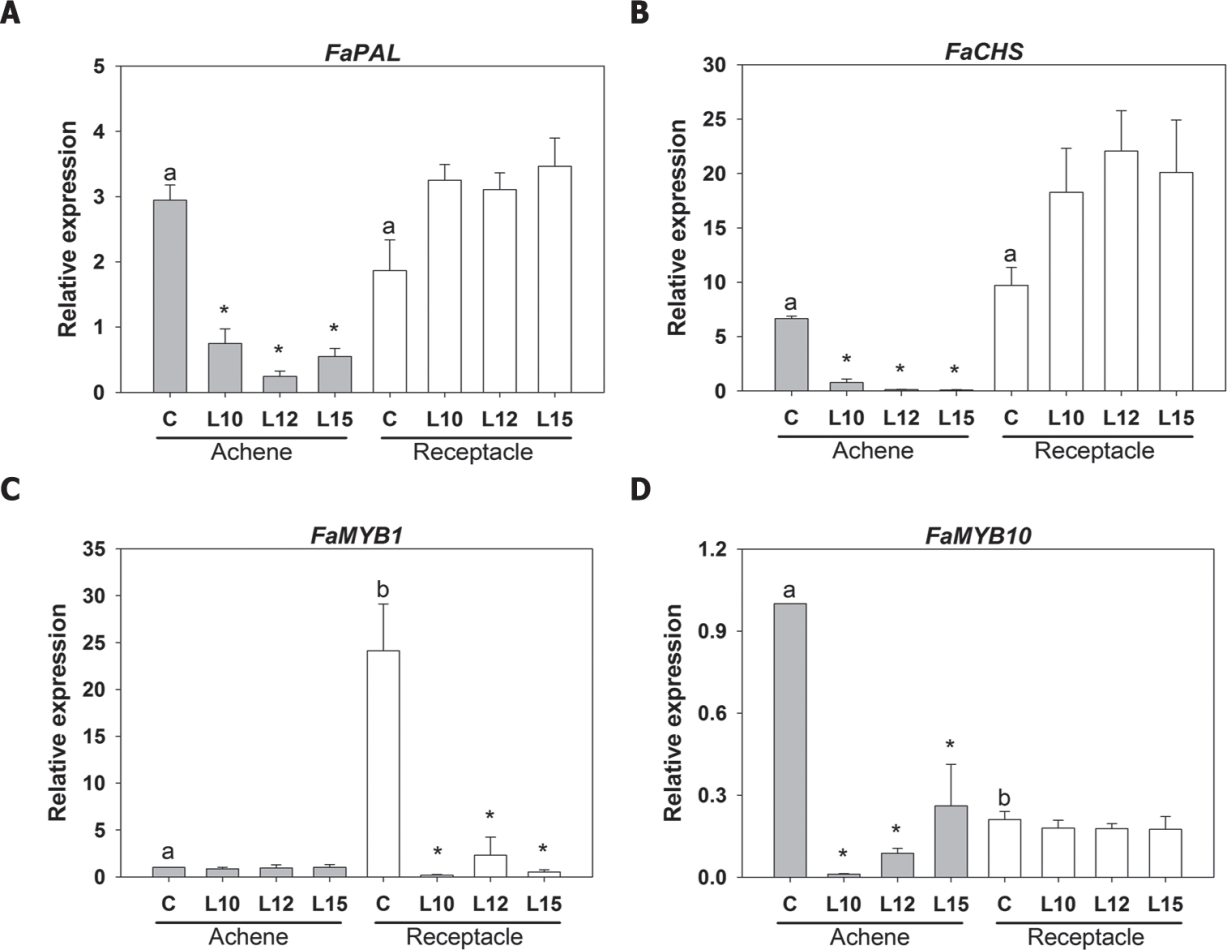
Relative expression of strawberry genes (*FaPAL*, *FaCHS*, *FaMYB1*, and *FaMYB1*), as determined by qRT–PCR, in the achenes and receptacles of ripe strawberry fruits. Asterisks indicate significant differences between the transgenic lines and the control for each sample using ANOVA and the Tukey HSD test adjusted to a 95% significance level. Different letters indicate significant differences within the control lines (achene and receptacle) for each gene using ANOVA and the Tukey HSD test adjusted to a 95% significance level.

The reduced redness in colour in the transgenic achenes is probably due to the diminished synthesis of flavonoids, which constitute the group of phenylpropanoids that accumulate in this organ during ripening (Fait *et al.*, 2008). In the biosynthesis of flavonoids, the expression of the genes encoding the key enzymes phenylalanine ammonia lyase (*PAL*) and chalcone synthase (*CHS*) plays a major role due to tight transcriptional regulation in this metabolic pathway (Salvatierra *et al.*, 2010; Gou *et al.*, 2011; Hichri *et al.*, 2011; Muñoz *et al.*, 2011). Therefore, the expression level of these genes was investigated in the control and transgenic achenes and receptacles during the red stage. As shown in Fig. 5A and B, no differences in the expression of *FaPAL* and *FaCHS* were found between the control and transgenic lines in the receptacles, which is consistent with the lack of visual differences between the control and transgenic receptacles. In fact, although no significant differences were found, a tendency for higher expression in the transgenic receptacle was observed (Fig. 5A, B). However, the expression of *FaPAL* and *FaCHS* was strongly reduced in the achenes of the transgenic fruits (Fig. 5A, B), which is consistent with their decreased pigmentation (Fig. 3A). Some genes of the *MYB* family have been reported to play a regulatory role in the flavonoid pathway. In the strawberry, the *FaMYB1* gene is suggested to be a transcriptional repressor in the regulation of anthocyanin biosynthesis (Aharoni *et al.*, 2001; Salvatierra *et al.*, 2013). The study of the relative expression of this gene in the achenes and receptacle of control and *etr1-1* transgenic ripe fruits showed a higher expression in the receptacle in comparison with the achene, and, as expected from its putative role in anthocyanin biosynthesis, it was significantly down-regulated in the transgenic receptacle (Fig. 5C). More recently, *FaMYB10* has been positively associated with colour development in strawberry fruits (Lin-Wang *et al.*, 2010). The analysis of the relative expression of this gene in the ripe fruits of the control and transgenic *etr1-1* lines showed that its expression was down-regulated only in the transgenic achenes (Fig. 5D), whereas the expression of this gene was >3-fold higher in the receptacle. The effect of ethylene on the expression of the two genes encoding the MYB transcription factors (*FaMYB1* and *FaMYB10*), as deduced from analysis of the *etr1-1* fruits, is in agreement with the changes in the expression of the *FaPAL* and *FaCHS* genes (Fig. 5A, B) and the less coloured phenotype of the transgenic achenes (Fig. 3A).

**Fig. 6.**
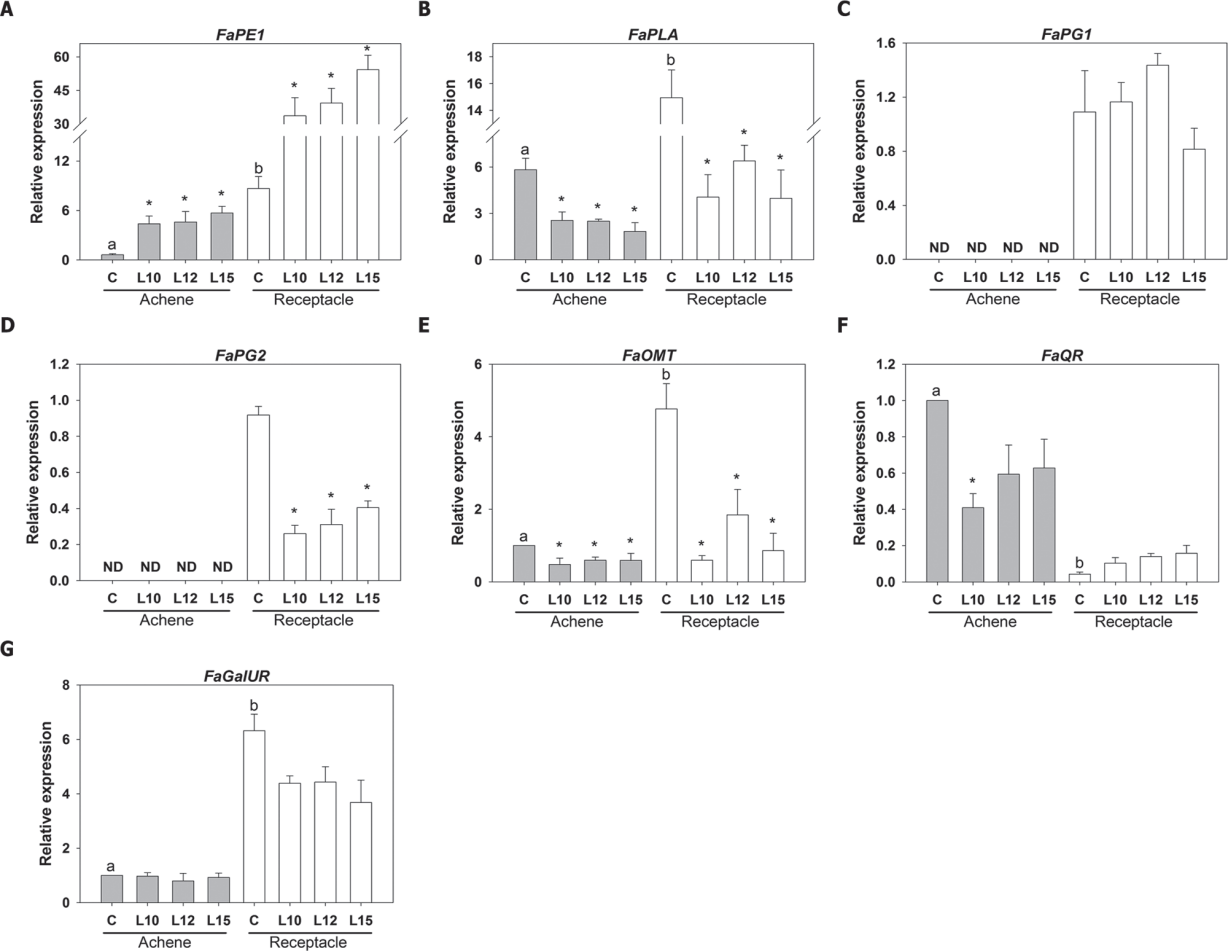
Relative expression of strawberry genes (*FaPE1*, *FaPLA*, *FaPG1*, *FaPG2*, *FaOMT*, *FaQR*, and *FaGalUR*), as determined by qRT–PCR, in the achenes and receptacles of ripe strawberry fruits. Asterisks indicate significant differences between the transgenic lines and the control for each sample using ANOVA and the Tukey HSD test adjusted to a 95% significance level. Different letters indicate significant differences within the control lines (achene and receptacle) for each gene using ANOVA and the Tukey HSD test adjusted to a 95% significance level.

A number of genes involved in cell wall restructuring and disassembly are canonically associated with fruit ripening (Rose and Bennett, 1999; Brummell, 2006). Therefore, to investigate the effect of altering the ethylene sensitivity on cell wall metabolism, the expression level of three cell wall-related genes was analysed in the achenes and receptacles of ripe fruits. The genes *pectin methyl esterase 1* (*FaPE1*) (Castillejo *et al.*, 2004), *pectate lyase A* (*FaPLA*) (Benitez-Burraco *et al.*, 2003), and *polygalacturonase 1* and *2* (*FaPG1* and *FaPG2*) (Quesada *et al.*, 2009) were selected. These genes have been shown to play important roles in the ripening of strawberry fruit through functional analyses (Osorio *et al.*, 2008; Santiago-Domenech et al, 2008; Quesada *et al.*, 2009). All of these pectin-depolymerizing genes showed higher expression in the receptacle than in the achenes in the control fruits, but ethylene was found to affect their expression in the two organs (Fig. 6A–D). *FaPE1* expression was enhanced in both the achenes and receptacle in the three transgenic lines (Fig. 6A), which is in agreement with its previously reported down-regulation by ethylene and induction by 1-MCP, an inhibitor of ethylene action (Castillejo *et al.*, 2004). In contrast, the diminished ethylene sensitivity of the transgenic lines caused a decrease in the expression of *FaPLA* in both the achenes and receptacles, indicating that this gene is positively regulated by ethylene. Interestingly, the *FaPG2* transcript was also down-regulated in the receptacle in the three transgenic lines (Fig. 6D), but no changes were observed in *FaPG1* expression (Fig. 6C). It has been described that these two *PG* genes are up-regulated during fruit ripening, but their expression patterns and cell wall targets are different (Quesada *et al.*, 2009). Given the different expression levels of the cell wall-related genes observed in the transgenic fruits, the firmness was next determined in ripe control and *etr1-1* fruits. Non-significant differences in firmness were observed (38.2±0.9, 37.1±0.8, 36.8±0.9, and 38.1±0.7g mm^–2^ in the control, L10, L12, and L15, respectively; values are the means ±SE).

The ripening of strawberry fruit is accompanied by the production of volatile compounds that account for the aroma of the fruit. Among these, furaneol is known to be important due to its high production by ripe fruits and its low odour threshold, quinone oxidoreductase (FaQR) being a key enzyme in its production (Raab *et al.*, 2006). Analysis of the expression of the *FaQR* gene in the control and transgenic *etr1-1* lines showed a non-significant decrease in the expression of FaQR in the achenes as a result of ethylene insensitivity (Fig. 6F), and the expression of this gene was significantly lower in the receptacle in all cases. Mesifurane is another important contributor to strawberry fruit aroma, which is controlled by *O*-methyl transferase (FaOMT) (Lunkenbein *et al.*, 2006; Zorrilla-Fontanesi *et al.*, 2012). Transcript analysis showed that the expression of *FaOMT* was down-regulated in the transgenic *etr1-1* lines compared with the control in both the achenes and receptacle (Fig. 6E). Additionally, the expression levels of the gene encoding d-galacturonate reductase (*FaGalUR*), which is involved in ascorbic acid biosynthesis, were not significantly different at the red stage in the transgenic lines in comparison with the control (Fig. 6G).

### Specific metabolites are differentially regulated in the achenes and receptacle of the *etr1-1* strawberry fruits

To investigate further the role of ethylene in fruit ripening, metabolic profiling of the primary metabolites was performed using GC-MS (Schauer *et al.*, 2006). The achenes and receptacles of the control and the three transgenic lines were analysed at the green, white, and red ripening stages (Supplementary Fig. S1 at *JXB* online).

**Fig. 7.**
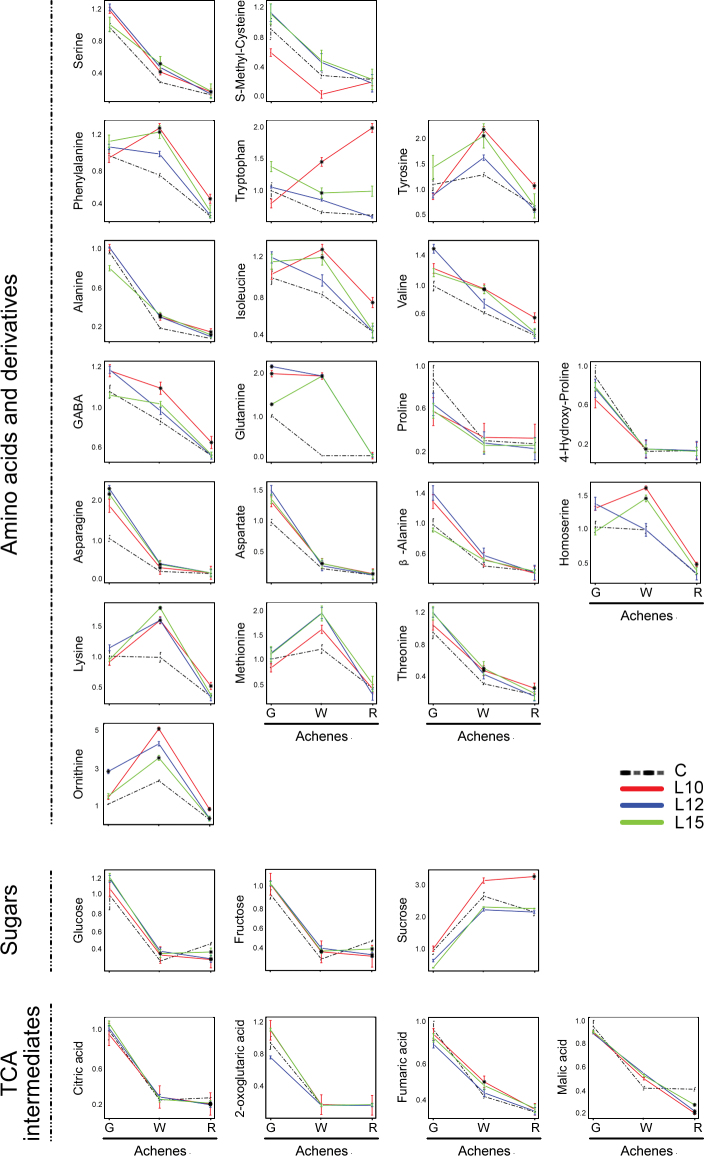

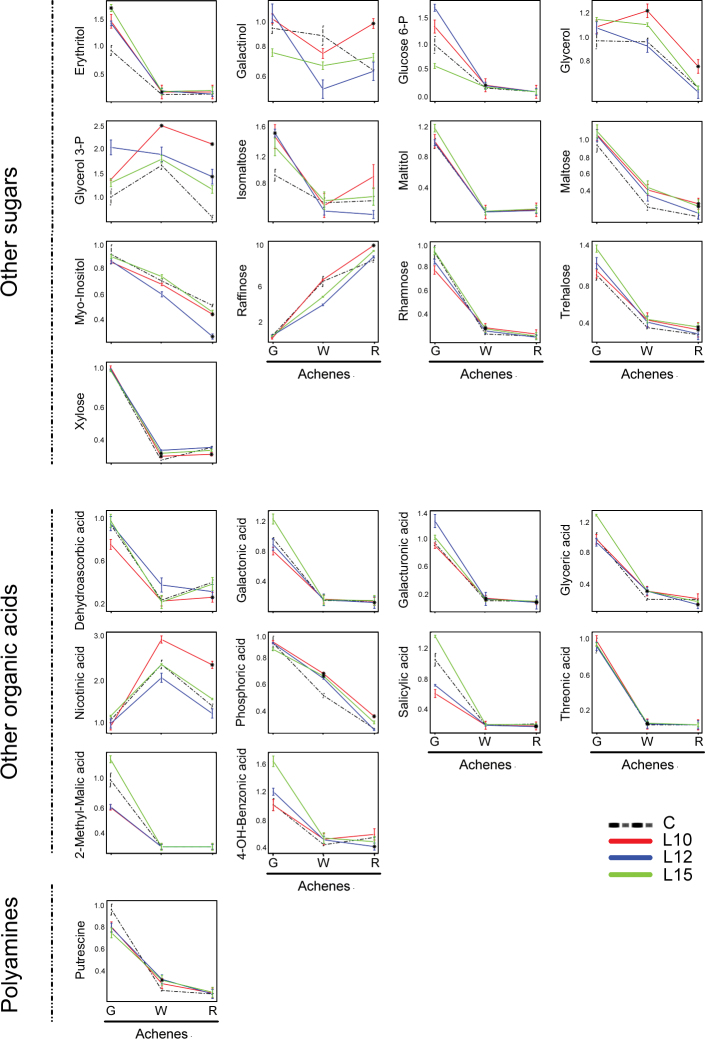
Primary metabolite levels in the achenes of the control (C) and *etr1-1* transgenic lines (L10, L12, and L15) at the three developmental stages (G, green; W, white; R, red). Data, calculated as described in the Materials and Methods, are relative to the value obtained for the control at the G stage. Values are the means ±SE of three replicates. Asterisks indicate significant differences (*t*-test, *P* < 0.01) between the transgenic lines and the control at the same developmental stage.

In the control achenes, the amino acid levels generally declined during ripening, as has been previously described (Fait *et al.*, 2008). While this pattern was conserved in the transgenic lines, it is important to note that most of the free amino acids were at higher levels in the achenes of the three transgenic lines than in the control (Fig. 7). Such differences were particularly prominent at the green stage for the N-rich amino acids asparagine and glutamine (three lines) and extended to most of the other amino acids in the white stage and, in some cases, also in the red stage. There are some amino acids that showed even higher accumulation from the green stage to the white stage in the transgenic achenes compared with the control. These include the two aromatic amino acids phenylalanine and tyrosine (L10 and L15), and the basic N-rich amino acids lysine and ornithine (L10 and L15).

The sugar and organic acid contents in the achenes were not altered in the transgenic lines compared with the control at the green stage. However, a decrease was observed in the levels of citric acid and malic acid at the red stage (all lines; Fig. 7). Similarly, all lines showed a decrease in the levels of the major sugars glucose and fructose at the red stage. Additionally, the *etr1-1* lines displayed an increase in the levels of maltose and trehalose at the red stage (lines 10 and 15; Fig. 7)

**Fig. 8.**
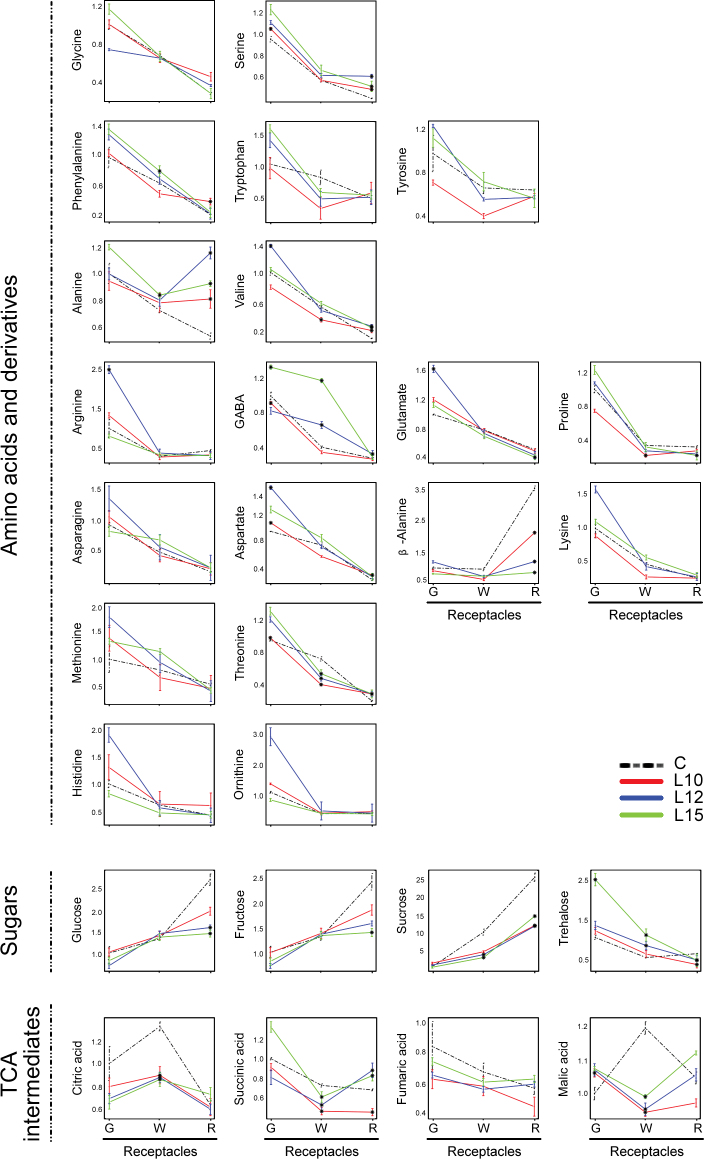

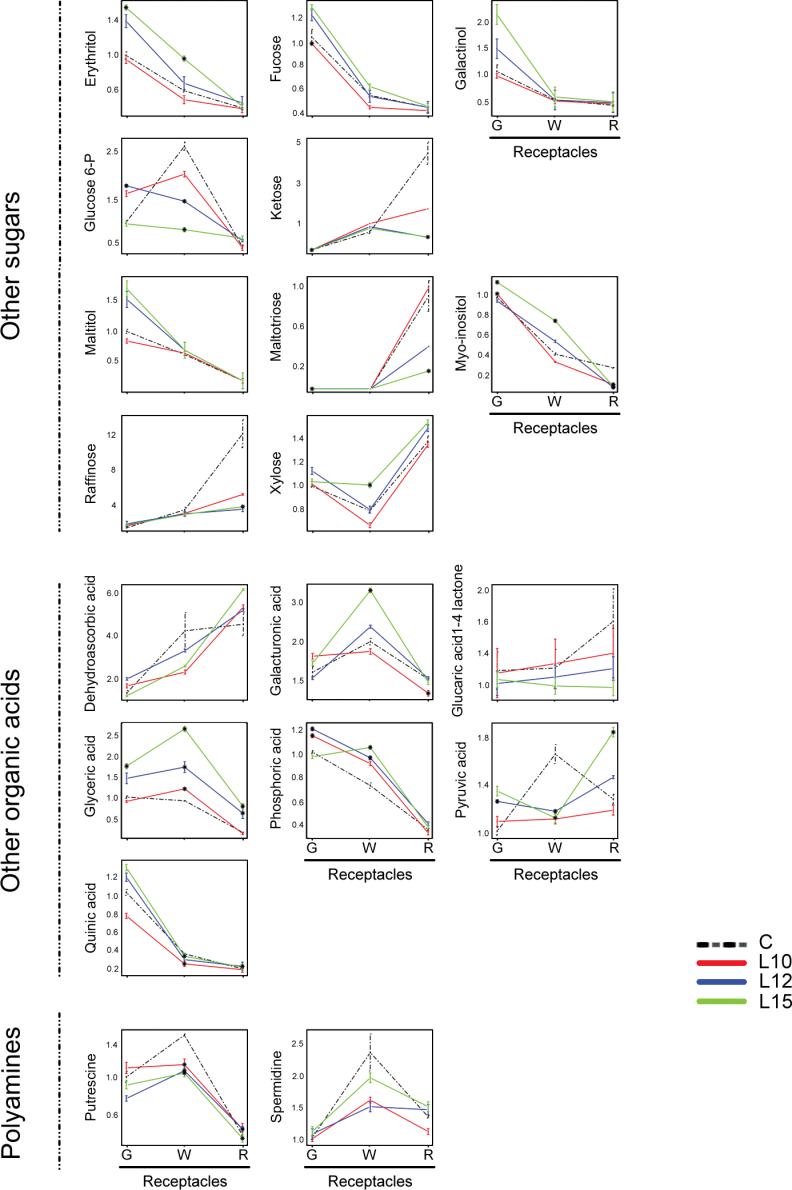
Primary metabolite levels in the receptacles of the control (C) and *etr1-1* transgenic lines (L10, L12, and L15) at the three developmental stages (G, green; W, white; R, red). Data, calculated as described in the Materials and Methods, are relative to the value obtained for the control at the G stage Values are the means ±SE of three replicates. Asterisks indicate significant differences (*t*-test, *P* < 0.01) between the transgenic lines and the control at the same developmental stage.

In the receptacle, the general analysis of the amino acid contents revealed that significant differences between the control and the three transgenic lines were rather limited, with only certain amino acids affected and only at specific stages (Fig. 8). A decrease in the threonine content was observed at the white stage (all three lines) and an increase at the red stage (all three lines). Similarly, an increase in the levels of alanine, valine, and serine (all three lines) was observed (Fig. 8).

Differences in the sugar and organic acid contents between the transgenic and control receptacles were also found at the white and red stages (Fig. 8). At the white stage, a significant reduction in the tricarboxylic acid (TCA) cylce intermediates citric acid, succinic acid, and malic acid (all lines) was observed compared with the control. This reduction was accompanied by a significant decrease in the most important sugars (glucose, fructose, sucrose, and trehalose) at the red stage (Fig. 8). The metabolic analysis also showed that the transgenic receptacles were characterized by decreases in glucose-6P (L12 and L15), pyruvic acid (all lines), and putrescine (all lines) at the white stage, as well as decreased levels of β-alanine at the red stage (all lines) (Fig. 8).

### Correlation analysis of metabolites in the achenes and receptacles

**Fig. 9.**
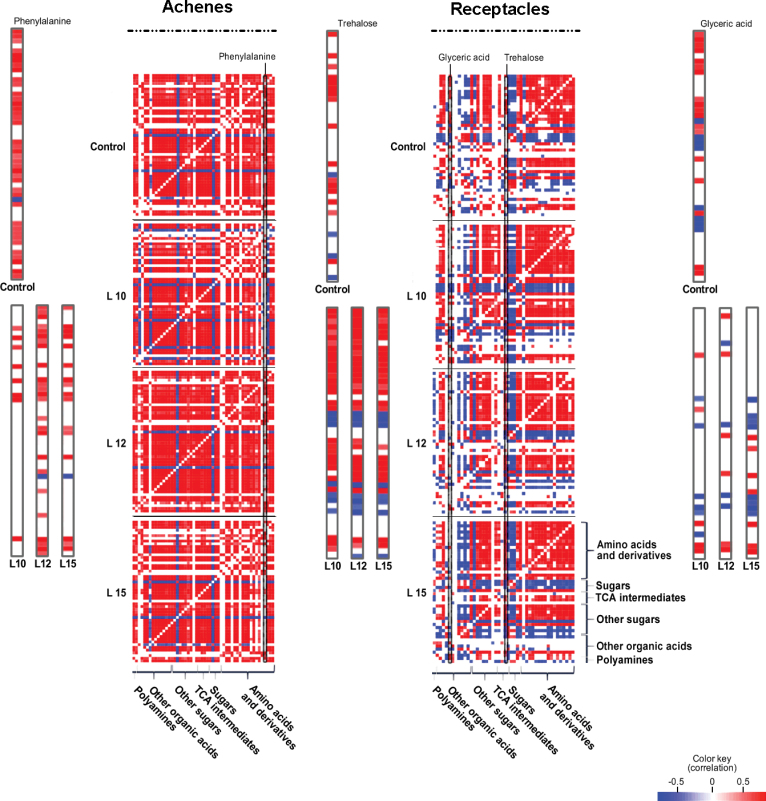
Visualization of metabolite–metabolite correlations. Heat maps of the metabolite–metabolite correlations across ripening stages (G, W, and R) for the achenes (A) and receptacles (B) of the control and transgenic lines are shown. The metabolites are grouped by compound class, and each square represents the correlation between the metabolite heading the column and the metabolite heading the row. Correlation coefficients and significances were calculated by applying the Pearson algorithm using R-environment. Here, only the significant correlations are presented (*P* < 0.05). Positive and negative correlations are presented in red and blue, respectively.

Next the coordinated metabolic changes were identified by performing a pair-wise correlation analysis using Pearson’s correlation at a strict stringency threshold (*P* < 0.05; values in Supplementary Tables S2–S9 at *JXB* online). This analysis reflects the degree of coordination of the metabolic changes in the corresponding organ. Here, this analysis was performed in the achenes and receptacles of each transgenic line and the control to identify any interference in the correlations caused by the diminished sensitivity to ethylene of the transgenic lines. However, it is important to note that any conclusions drawn from these data must be made cautiously, given the low number of time points used in this study. In general, it was observed that the total number of correlations was higher in the achenes than in the receptacles (Fig. 9). The number of correlations in the achenes was 963 in the control, and 933, 1030, and 897 correlations were observed in L10, L12, and L15, respectively. In the receptacle, 559 correlations were observed in the control, and 638, 558, and 650 were observed in L10, L12, and L15, respectively. The results for the control plants were anticipated because they agree with previous reports (Fait *et al.*, 2008). However, it is interesting that the transgenic lines displayed very similar coordination. Focusing on specific changes associated with the transformation event revealed some interesting changes. For example, in the achenes, the most conspicuous change was the loss of the positive correlation of phenylalanine with most of the metabolites in the transgenic lines compared with the control (Fig. 9). This is most probably due to the down-regulation of *FaPAL* in the transgenic lines (Fig. 5A). Regarding the receptacles, there were two dramatic changes in the transgenic lines (Fig. 9). The first is the loss of the strong positive correlation of glyceric acid with the majority of the amino acids in the transgenic lines. This is probably due to the dramatically enhanced content of 3C-derived amino acids (serine, alanine, and valine) in the transgenic lines. Secondly, trehalose was negatively correlated with glucose, fructose, sucrose, fumaric acid, and glucose-6P, and was positively correlated with amino acids in the transgenic lines. This observation is in keeping with the main role hypothesized for this disaccharide as a result of changes in carbon allocation between the sink and source tissues (Eveland and Jackson, 2012). However, given that trehalose-6-phosphate has been proposed as a signalling molecule whose metabolism is regulated by sucrose (Ma *et al.*, 2011), it cannot be disregarded that it mediates the changes observed in the sucrose content in the transgenic receptacles.

## Discussion

The strawberry fruit has traditionally been considered to be non-climacteric because its ripening process does not follow the characteristic ethylene production and respiration of the so-called climacteric fruits (Given *et al.*, 1988). However, changes in the endogenous production of ethylene, albeit very small, have been reported in the intact fruit (Iannetta *et al.*, 2006). In addition, there are several reports showing that some molecular responses associated with fruit development are altered by treatment with ethylene, ethylene precursors, or ethylene inhibitors (Trainotti *et al.*, 2001; Castillejo *et al.*, 2004; Bustamante *et al.*, 2009: Villareal *et al.*, 2009, 2010). These data suggest a role for ethylene in fruit development and/or ripening. However, there are several aspects of the strawberry fruit that must be taken into consideration. First, the strawberry fruit is composed of two organs that are very different in terms of origin, physiological role, and prevailing metabolic networks; these organs are the achenes, which are the true fruits, and the receptacles, which are derived from the flower receptacles (Fait *et al.*, 2008; Csukasi *et al.*, 2011). Analysis of the complete berry, which includes both parts, makes it difficult to interpret the results obtained due to the heterogeneity of the sample. Secondly, the relative contribution of each organ to the whole berry is highly dependent on the developmental stage of the fruit. While the relative contribution of the achenes to the biomass of the whole fruit is high in green fruits, this contribution is considerably smaller in red fruits (see Supplementary Fig. S1 at *JXB* online). A third consideration is the inherent difficulty in measuring the independent endogenous production of ethylene in the achenes and receptacle, which is exacerbated by the low level of internal production of this hormone by the strawberry fruit (Iannetta *et al.*, 2006). Therefore, the strategy used here for overcoming these issues was to generate transgenic lines with reduced sensitivity to endogenous ethylene and to perform separate analyses of the achenes and receptacles. These aims were achieved by overexpressing the mutant *Arabidopsis* allele *etr1-1*, as exhaustive studies on the function and interaction of the various ethylene receptors in *Arabidopsis* have demonstrated that the dominant mutant receptor gene *etr1-1* prevents the ethylene response independently of other wild-type receptors (Liu and Wen, 2012). This means that the transgenic strawberry *etr1-1* lines are partially insensitive to ethylene, which was apparent from its phenotype (Fig. 2), despite the occurrence of other active endogenous ethylene receptors. Interestingly, the endogenous ethylene receptors were down-regulated in the transgenic lines, which agrees with the positive response to ethylene treatment reported for these three genes in ripe fruits (Trainotti *et al.*, 2005).

### Full ripening of the achene requires ethylene action

The expression of some of the key genes involved in ethylene biosynthesis (i.e. *FaACS3*, *FaACS4*, and *FaACO3*) was highest in the green achenes. A previous proteomic study in achenes identified *S*-adenosylmethionine synthetase as one of the most abundant proteins in green achenes (Aragüez *et al.*, 2013). This enzyme is involved in the biosynthetic ethylene pathway (Yang and Hoffman, 1984). All of this information suggests that ethylene production is important in the achene at this stage, a hypothesis that is supported by the changes in gene expression and metabolites that were observed when the ethylene action was reduced in the *etr1-1* transgenic plants. This stage corresponds to fruits at 12 d post-anthesis, at which time the embryo is in the cotyledon stage and the dry pericarp of the achene is developing. This is consistent with the role proposed for ethylene during early seed development (Lombardo *et al.*, 2011). Later developmental changes are dominated by the function of the mature achenes as dispersal units, namely lignification, the synthesis of secondary defence metabolites, and the accumulation of storage compounds (Aharoni and O’Connell, 2002; Fait *et al.*, 2008). The key genes involved in the biosynthesis of phenolics and flavonoids, *FaPAL* and *FaCHS*, showed reduced expression in the *etr1-1* achenes, indicating a regulatory role for ethylene in the synthesis of these compounds. Consistent with this, a visible outcome was the diminution of coloured flavonoids in the mature achene, which also showed a lower content of phenolics. The fact that regulatory *FaMYB10* is significantly down-regulated in transgenic achenes indicates that this gene might be involved in the ethylene signalling pathway in this organ.

There were significant changes in the levels of the primary metabolites. The sharp decline in the free amino acid content, which characterizes the ripening of the achenes (Fait *et al.*, 2008), was somewhat altered in the three transgenic lines; the free amino acid level was higher than in the control. This difference might result from the impaired synthesis of storage proteins in the transgenic achenes. The higher accumulation of phenylalanine and tyrosine in the white achenes of the transgenic lines was most probably due to the diminished synthesis of phenylpropanoids, which was caused by the down-regulation of *FaPAL* and *FaCHS*. There is also a significant increase of lysine in transgenic achenes at the white stage. Since this amino acid is in the pathway leading to the synthesis of ethylene, this might be indicative of an altered ethylene biosynthetic pathway in transgenic achenes. When analysing the correlations between metabolites, the positive correlation between phenylalanine and most of the analysed metabolites was absent in the transgenic lines. Ethylene was also important for normal energy metabolism in the achenes. Thus, the effects of diminished sensitivity to this hormone were also evident in the red stage, which exhibited lower levels of sugars, such as glucose, frructose, and sucrose, as well as TCA cycle intermediates, such as citric acid and malic acid. The late stages of achene maturation are characterized by a depletion in the metabolites of the major energy pathways (Fait *et al.*, 2008), an event that was enhanced in the *etr1-1* achenes.

Taken together, the present results demonstrate that ethylene is involved, either directly or indirectly, in the developmental changes that occur in both the early and late developmental stages of the achenes. Its involvement in the ripening of the achenes appears critical for phenylpropanoid metabolism and strawberry aroma production. However, the presence of a dry pericarp reveals that the major changes that take place during the ripening of this organ, such as the accumulation of storage and protective compounds, are not comparable with those of fruits with a fleshy pericarp. Therefore, despite the fact that the achene is a true fruit, given the low endogenous production of ethylene by the whole fruit (Iannetta *et al.*, 2006) and its lack of involvement in some changes that are commonly associated with the ripening of fleshy fruits (Seymour, 1993), it cannot be considered a canonical climacteric fruit.

### Ripening of the receptacle partially resembles the climacteric ripening of fleshy fruits

Although the fleshy part of the strawberry is not a true fruit because it does not develop from the ovary and is the result of the enlargement of the flower receptacle, it is important for seed dispersal and human consumption. This functional evolutionary convergence with true fleshy fruits reflects that, despite some differences, changes in the receptacle during ripening, such as those related to cell wall disassembly as well as primary and secondary metabolism, are similar to those of true fruits (Castillejo *et al.*, 2004; Rosli *et al.*, 2004; Fait *et al.*, 2008; Quesada *et al.*, 2009; Osorio *et al.*, 2011).

The highest expression of *FaACS1* occurred at the green/white stage, when cell expansion is very active and just before the changes associated with the ripening of this tissue are triggered. In the tomato, the sequential expression of the members of the *ACS* and *ACO* gene families during fruit development has been reported (Cara and Giovannoni, 2008; Yokotani *et al.*, 2009). Some of these genes are associated with the early production of ethylene (*SlACS1a* and *SlACS6*), while others are associated with the climacteric production of this hormone (*SlACS2* and *SlACS4*). Interestingly, the protein sequence analysis showed the grouping of FaACS1 with SlACS2 and SlACS4, indicating a parallelism between ripening in the strawberry receptacle and the tomato fruit.

Changing the ethylene sensitivity resulted in considerable changes in the levels of the ethylene receptor genes *FaETR1*, *FaETR2*, and *FaERS1*, whose expression in the control fruits was higher in the receptacle than in the achenes. Previous studies in the tomato showed a significant down-regulation of transcripts related to ethylene perception in the *nor* (non-ripening) mutant, in which ripening is almost completely inhibited (Osorio *et al.*, 2011). Additionally, in the receptacle, changes in the expression of some pectin-related genes were observed. The involvement of these genes in cell wall disassembly at the ripe fruit stage has been previously reported (Jiménez-Bermúdez *et al.*, 2002; Castillejo *et al.*, 2004; Quesada *et al.*, 2009). It was found that *FaPE1* was up-regulated while *FaPLA* was down-regulated in the transgenic receptacles. Interestingly, the peaks of highest expression for these two genes during strawberry development and ripening are not coincident (Benitez-Burraco *et al.*, 2003; Castillejo *et al.*, 2004). This might explain the opposite effects for these two genes in the transgenic receptacle in this study, which resulted from nullifying/mitigating the action of endogenous ethylene, which was produced at specific developmental stages. In addition, the expression of *FaPG2* but not *FaPG1* was altered in the transgenic receptacles, indicating that ethylene does not have a general effect on all ripening-associated genes. This explains why no effect was found on fruit firmness, which is controlled by the coordinated action of all cell wall-degrading enzymes.

The metabolic programme associated with receptacle ripening is also regulated by ethylene. Decreasing ethylene perception caused a lower content of TCA cycle intermediates, including citric acid, succinic acid, and malic acid, at the white stage. It is possible that this is a consequence of the down-regulation of the alternative respiratory pathway because this pathway has demonstrated sensitivity to changes in ethylene in other plant systems (Wang *et al.*, 2010). The lower content of TCA cycle intermediates at the white stage is accompanied by a higher content of some amino acids, such as serine, phenylalanine, and alanine, and a diminished content of the major sugars fructose, glucose, and sucrose in the red transgenic receptacle compared with the control. Interestingly, similar changes in sugars and TCA cycle intermediates have also been observed during the ripening of two ripening mutants of the tomato, *nor* and *Nr*, in comparison with the wild-type fruit (Osorio *et al.*, 2011). The *Nr* gene mutation results in the expression of an ethylene receptor with an impaired ability to bind ethylene (Lanahan *et al.*, 2009), while the *nor* mutation has been suggested to be involved in ethylene biosynthesis (Tigchelaar *et al.*, 1973). These orchestrated metabolic changes found in the strawberry receptacle are expected due to the connectivity reported between the different metabolic pathways in the ripening receptacle (Fait *et al.*, 2008). Analysis of correlations among metabolites in the receptacles identified specific changes that were common to all transgenic lines in comparison with the control. Independently of their metabolic significance, which deserves further study, these changes reveal a role for ethylene in the metabolism of the ripening receptacle. This role most probably extends to the production of strawberry aroma compounds. Although the production of volatiles was not evaluated in the transgenic receptacles, the decreased expression of *FaOMT* supports a change in mesifurane production. It has been reported that natural variation of mesifurane is associated with the expression of the *FaOMT* gene (Zorrilla-Fontanesi *et al.*, 2012).

The analysis of mutants that are affected by ethylene signalling has been a useful tool to investigate ethylene-dependent or ethylene-independent processes during tomato ripening at the systems biology level (Osorio *et al.*, 2011). In the strawberry, there are no previous reports on the occurrence of mutants in ethylene synthesis and/or signalling. However, the transformation of the strawberry with the ethylene receptor allele *etr1-1* and the subsequent dominant-negative effect in the ethylene perception have allowed for the examination of the role of ethylene in strawberry fruit ripening. The data presented here indicate that ethylene is required for the normal development of the strawberry fruit, in which it acts differently in the achenes and receptacle. In achenes, it acts at the green and red stages, while in the receptacle it acts at the green/white stages. In both organs, ethylene selectively affects the expression of genes involved in ethylene reception, phenylpropanoid metabolism, cell wall degradation, and strawberry aroma production. Further dissection of its differential regulatory role in the two organs would require a more exhaustive time and cell type analysis. However, the strawberry fruits cannot be considered as canonical climacteric fruits. Finally, this study supports the need to study the growth and ripening process of these two strawberry fruit components independently to answer key questions, such as the role of hormones. The two organs follow parallel yet distinctive developmental programmes (Fait *et al.*, 2008; Csukasi, 2012), and their connectivity at early developmental stages has been known for a long time (Nitsch, 1950). However, this relationship remains to be further explored during ripening. Such an exploration must be extended to the role played by other hormones, such as abscisic acid (ABA), whose involvement in strawberry fruit ripening has recently been reported (Chai *et al.*, 2011; Jia *et al.*, 2011). This result is particularly interesting due to the connections that were previously established between ethylene and ABA signalling. For example, in grape berry ripening, a sequential action has been proposed for ethylene and ABA in the regulation of the ripening process (Ziliotto *et al.*, 2012). More interestingly, in the promoter of the *FaOMT* gene, which was found here to be down-regulated upon diminution of ethylene sensitivity, an ABA response element was identified (Zorrilla-Fontanesi *et al.*, 2012).

## Supplementary data

Supplementary data are available at *JXB* online.

Figure S1. Photographs of fruits at the three studied ripening stages (G, green; W, white; R, red). Cross-sections of the receptacles at these three stages and of the achenes at the green stage are shown.

Figure S2. Screening of transgenic lines expressing the *etr1-1* gene under the control of the CaMV *35S* promoter.

Table S1. List of the primers used in the qRT–PCRs.

Table S2. Pearson correlation coefficients between the metabolite contents in the three ripening stages of control strawberry achenes.

Table S3. Pearson correlation coefficients between the metabolite contents in the three ripening stages of L10 strawberry achenes.

Table S4. Pearson correlation coefficients between the metabolite contents in the three ripening stages of L12 strawberry achenes.

Table S5. Pearson correlation coefficients between the metabolite contents in the three ripening stages of L15 strawberry achenes.

Table S6. Pearson correlation coefficients between the metabolite contents in the three ripening stages of control strawberry receptacles.

Table S7. Pearson correlation coefficients between the metabolite contents in the three ripening stages of L10 strawberry receptacles.

Table S8. Pearson correlation coefficients between the metabolite contents in the three ripening stages of L12 strawberry receptacles.

Table S9. Pearson correlation coefficients between the metabolite contents in the three ripening stages of L15 strawberry receptacles.

Supplementary Data
