# Supplementary material for: Ethylene is involved in strawberry fruit ripening in an organ-specific manner
Source: J Exp Bot. 2013 Oct 5;64(14):4421–39. doi: 10.1093/jxb/ert257 (PMC3808323; doi:10.1093/jxb/ert257)
Supplement: Supplementary Data [file supp_64_14_4421__index.html]

Ethylene is involved in strawberry fruit ripening in an organ-specific manner — Ethylene is involved in strawberry fruit ripening in an organ-specific manner — Supplementary Data 

# Ethylene is involved in strawberry fruit ripening in an organ-specific manner

## Supplementary Data

Data files

**Files in this Data Supplement:**

- Supplementary Data - Supplementary Data
- Supplementary Data - Supplementary Data
