# Supplementary material for: Ethylene is involved in strawberry fruit ripening in an organ-specific manner
Source: J Exp Bot. 2013 Oct 5;64(14):4421–39. doi: 10.1093/jxb/ert257 (PMC3808323; doi:10.1093/jxb/ert257)
Supplement: Supplementary Data [file supp_ert257_jexbot100586_file001.pdf]

Supplementary Data

Analysis of the role of ethylene in strawberry fruit ripening using ethylene-insensitive plants

Catharina Merchante, José G. Vallarino, Sonia Osorio, Irene Aragüez, Natalia Villarreal, María Ariza, Gustavo Fernández, Nieves Medina-Escobar, Marcos Civello, Alisdair R. Fernie, Miguel A. Botella, Victoriano Valpuesta.

Figure S1.

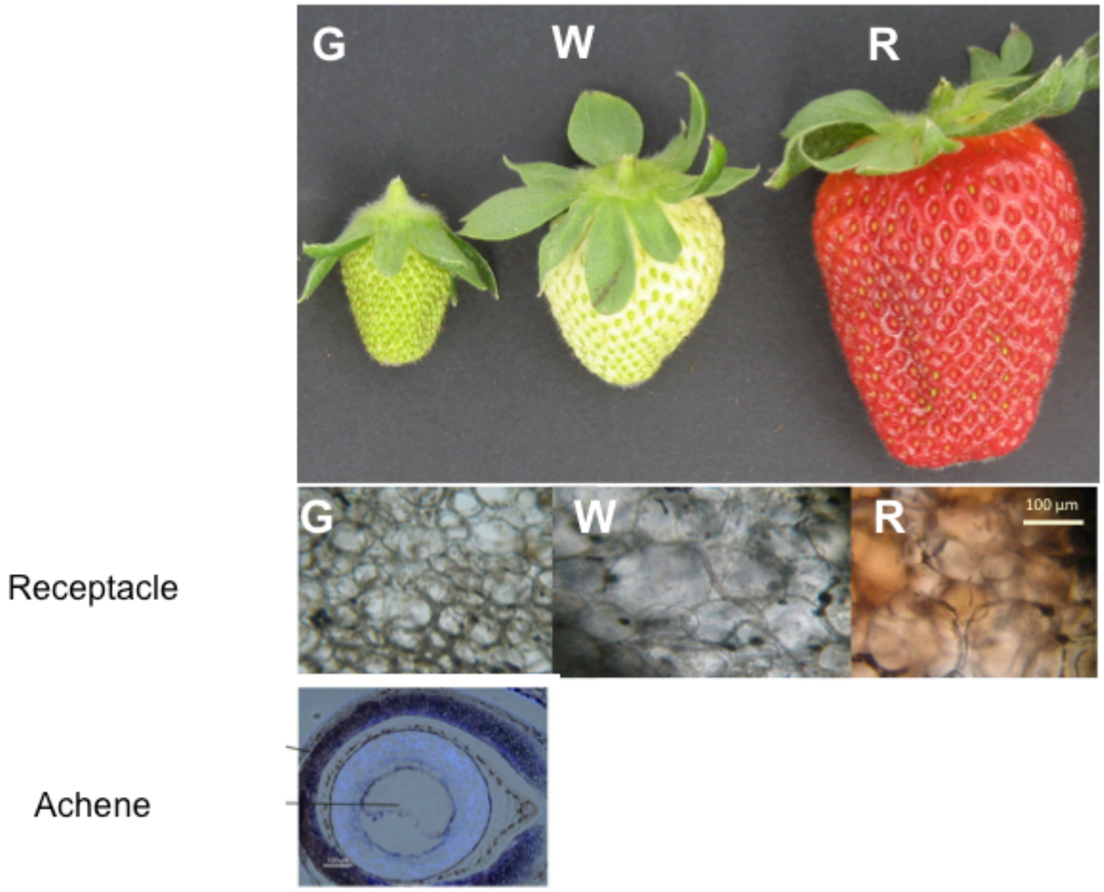

Illustration of strawberry fruit developmental stages selected for the studies. **A.** Photographs of representative fruits named as green (G), white (W) and red (R) stages of development. **B.** Sections of receptacle at the same stages (G, W, R) showed in A. **C.** Cross-section of the achene corresponding to the fruit at the G stage showed in A.

Figure S2.

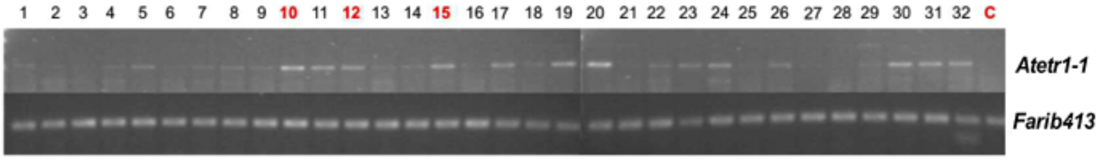

Expression of *Atetr1-1* by RT-PCR in 32 independent transgenic lines expressing the Arabidopsis gene. In red colour are indicated the lines selected for further studies.

Supplemental Table 1. List of primers

AtETR1	F (5´GTCGTGCCAACTGGGAG 3´)<br>R (5´GGAGAGCGATTTGGTAGTTT 3´)
FaACS1	F (5´ CTTACCATCCCACCACCAAC 3´)<br>R (5´ TTCTTTTGCTCCTTGTGCTG 3´)
FvACS2	F (5´ GATCCTGATCGTGTAGTCATGG 3´)<br>R (5´ GTTGTTTCGGGCCTTTTCG 3´)
FvACS3	F (5´ ACCCGAAGGTGGTTTCTGC 3´)<br>R (5´ GATGCCGGAGTGTTTCAACT 3´)
FvACS4	F (5´ CCAACTTGGGATCAAAAGGA 3´)<br>R (5´ CCGACTTGGGTCAAACTTAGC 3´)
FaACO1	F(5´ TACCTCAAGCACCTTCCTCGC 3´)<br>R (5´ TTAGTGCCAAAGGTAGGACTA 3´) (Trainotti et al., 2005)
FaACO2	F (5´GAAAGCACCTTCTTCTTGCG 3`)<br>R (5´GCCTTAGCAGCCGTCATAAA 3´) (Trainotti et al., 2005)
FvACO3	F (5´CCTTCTTGATGACAATGAGTGG 3´)<br>R (5´CACCAACTCGGGTTGAGGAC 3´)
FaETR1	F (5’GGTGACCTCATTCCCGTCTTT 3’)<br>R (ACAGGCCTCCATCAGAATTGA 3’) (Trainotti et al., 2005)
FaETR2	F (5’GTTCGGCGTTGTTTTTAC 3’)<br>R (5’AGCTTCCCTCGTCGTCAC 3’) (Trainotti et al., 2005)
FaESR	F (5’CTTCAAGAGATTGGCGACCAC 3’)<br>R (5’GGATCCATTTCTGGGCTGAG 3’) (Trainotti et al., 2005)
FaPAL	F (5´GATGCAAAGGCTAAGGCAAG 3´)<br>R (5´AGCCCTAACGCTCTCAACCT3`) (Muñoz et al., 2011)
FaCHS	R (5´ GCCTTTGTTTGAGCTGGTCT 3`)<br>F (5´ CCCAGGAACATCTTTGAGGA 3´)(Muñoz et al., 2011)
FaMYB1	F (5’GGCGTGGTCGATCCAAGA 3’)<br>R (5’GCAACCTTCGCCGTGTTTT 3’)
FaMYB10	F (5’TTACCAACAGAACCACCACAGA 3’)<br>R (5’CCTCTAAACCAAGACCAGAACACA 3’) (Lin-Wang et al., 2010)
FaPE1	R (5´ CAAGTGCACCCAACTTCTGA 3`)<br>F (5´ CTGAGCTACCACAACGTCCA 3´)(Osorio et al., 2008)

FaPLA	R (5´ ACTTGGATGCCGCAGAGGA 3`)<br>F (5´ GAGGTGGGAGGGAAATGG 3´) (Benitez-Burraco et al., 2003)
FaPG1	R (5´ TGGAGGAGTTGGAGATGGAAA 3`)<br>F (5´ AGAGTGAAATGGCTGGTGAGG 3´)
FaPG2	R (5´ GCTCCTGGTGACTTTGATGTG 3`)<br>F (5´ CTCTACTTGGCGTTGTTGCTG 3´)
FaOMT	F (5'GCAGTTCTTGATGGTGGGATT 3')<br>R (5'ATGGTAATGGTGGAGTGGTCAG 3')
FaQR	F (5'AGAACCTTGGGTGTTGATTTGG 3')<br>R (5'TTGTTACTACCTTCCCGCCTTC 3') (Raab et al., 2006)
FaGalUR	F (5'CTGTAATCGGCATGGGAACT 3')<br>R (5'TGATGAGTTGGAGACGGAGA 3')
FaGAPDH	F (5'TCCATCACTGCCACCCAGAAGACTG 3')<br>R (5'AGCAGGCAGAACCTTTCCGACAG 3') (Opazo et al 2010)

References

Benítez-Burraco A, Blanco-Portales R, Redondo-Nevado J, Bellido ML, Moyano E, Caballero JL, Muñoz-Blanco J. 2003. Cloning and characterization of two ripening-related strawberry (Fragaria x ananassa cv. Chandler) pectate lyase genes. *Journal of Experimental Botany* **54,** 633-645.

Lin-Wang L, Bolitho K, Grafon K, Kortstee A, Karunairetnam S, McGhie T, Espley R, Hellens R, Allan A. 2010. An R2R3 MYB transcription factor associatedwith regulation of the anthocyanin biosyntheticpathway in Rosaceae. *BMC Plant Biology* **10,** 50.

Muñoz C, Sánchez-Sevilla JF, Botella MA, Hoffmann T, Schwab W, Valpuesta V. 2011. Polyphenol composition in the ripe fruits of Fragaria species and transcriptional analyses of key genes in the pathway. *Journal of Agricultural and Food Chemistry* **59,** 12598-12604

Opazo M, Figueroa C, Henriquez J, Herrera R, Bruno C, Valenzuela P, Moya_leon M. 2010. Characterization of two divergent cDNAs encoding xyloglucan endotransglycosylase/hydrolase (XTH) expressed in Fragaria chiloensis fruit. *Plant Science* **179,** 479-488.

Raab T, Lopez-Raez JA, Klein D, Caballero JL, Moyano E, Schwab W, Muñoz-Blanco J. 2006 FaQR, required for the biosynthesis of the strawberry flavor compound 4-hydroxy-2,5-dimethyl-3(2H)-furanone, encodes an enone oxidoreductase. Plant Cell **18:** 1023-1037.

Osorio S, Castillejo C, Quesada MA, Medina-Escobar N, Brownsey GJ, Suau R, Heredia A, Botella MA, Valpuesta V. 2008. Partial demethylation of

oligogalacturonides by pectin methyl esterase 1 is required for eliciting defence responses in wild strawberry (Fragaria vesca). *Plant Journal* 5**4,** 43-55.

Trainotti L, Pavanello A, Casadoro G. 2005. Different ethylene receptors show an increased expression during the ripening of strawberries: does such an increment imply a role for ethylene in the ripening of these non-climacteric fruits? *Journal of Experimental Botany* **56,** 2037-2046.
